# Use of selective visceral angiography in surgical strategy planning for celiac artery aneurysm in the celiacomesenteric trunk

**DOI:** 10.1186/s13019-024-02483-7

**Published:** 2024-01-19

**Authors:** Takasumi Goto, Hironobu Fujimura, Takashi Shintani, Takashi Shibuya, Shigeru Miyagawa

**Affiliations:** 1 Department of Cardiovascular Surgery, Toyonaka Municipal Hospital, 4-14-1, Shibahara, Toyonaka, Osaka 560-8565 Japan; 2 Department of Cardiovascular Surgery, Nippon Life Hospital, Osaka, Japan; 3 Department of Cardiovascular Surgery, Osaka University Graduate School of Medicine, Osaka, Japan

**Keywords:** Celiac artery aneurysm, Celiacomesenteric trunk, Collateral circulation, Selective visceral angiography

## Abstract

**Background:**

The celiacomesenteric trunk (CMT) is a common duct of the celiac artery (CA) and the superior mesenteric artery originating from the aorta, which is an uncommon anatomical variant of visceral artery circulation. Because of the variety of visceral circulation in those with CMT, the visceral circulation associated with each branch should be evaluated prior to surgical treatment of visceral artery aneurysm in the CMT.

**Case presentation:**

A 64-year-old woman was diagnosed with a CA aneurysm in the CMT. Aneurysmectomy of the aneurysm was performed successfully. On preoperative selective visceral angiography, the CA was seen to bifurcate into the common hepatic and splenic artery. The left gastric artery was directly isolated from the aorta and perfused to the common hepatic and splenic artery through collateral circulation. These findings showed that celiac artery embolization is anatomically feasible, even in cases of celiac artery aneurysm rupture.

**Conclusions:**

Selective visceral angiography can contribute to surgical strategy planning for CA aneurysm with CMT.

**Supplementary Information:**

The online version contains supplementary material available at 10.1186/s13019-024-02483-7.

## Background

The celiacomesenteric trunk (CMT), which consists of the common duct of the celiac artery (CA) and the superior mesenteric artery (SMA) branches of the aorta, is an uncommon anatomical anomaly of the visceral artery, with an incidence rate estimated to be under 0.5% [1–3]. The distal perfusion of the CMT into the CA and SMA is varied [1, 3]. Therefore, precise analysis of the visceral circulation is useful for planning and selecting the most appropriate surgical strategy, particularly in patients with a CA aneurysm (CAA) in the CMT. We report a successful case of aneurysmectomy for CAA in a patient with a CMT; the surgery was carefully planned using selective visceral angiography findings.

## Case presentation

A 64-year-old woman, with medical history of hypertension that was treated with amlozin (5 mg, Sumitomo Pharma, Japan) and azilsartan (20 mg, Towa, Japan), was initially admitted to another institution due to temporary back and abdominal pain. Her abdominal ultrasonography raised suspicion of CAA. Consequently, she was referred to our institution for further evaluation and treatment of CAA.

**Fig. 1:**
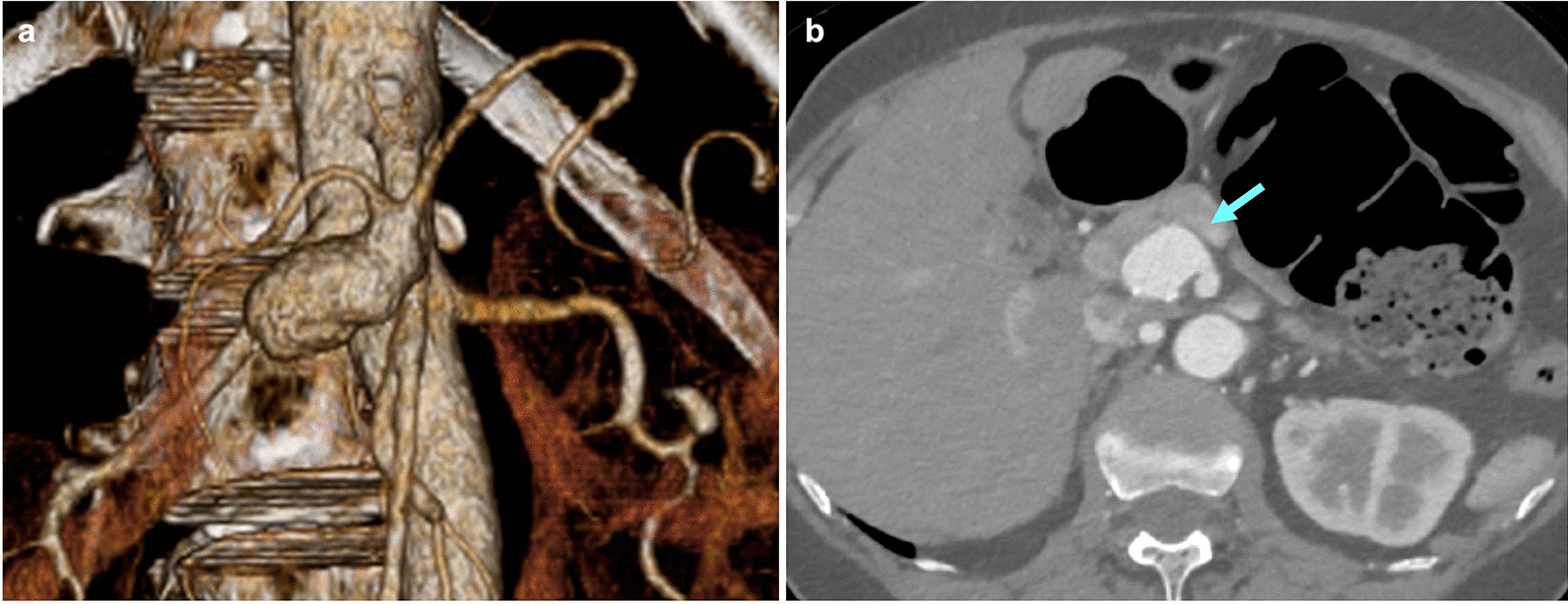
Preoperative enhanced computed tomography findings. A celiac artery aneurysm (shown by the blue arrow) located distal to the celiac artery branched from the celiacomesenteric trunk [panels (**a**) and (**b**) showing 3D and axial views, respectively]

At the time of presentation at our institution, she was asymptomatic, and her laboratory results were within the normal range (Additional file 1). Enhanced computed tomography (CT) revealed that the CA and SMA converged into the aorta via the CMT. The CAA was a saccular aneurysm and located approximately at the median of the CA, with a diameter of 26 × 28 mm (Fig. 1). Notably, no significant stenosis was present on the CMT. The patient was diagnosed with CAA, complicated by CMT. She was a good candidate for surgery since her CAA was > 20 mm [3].

**Fig. 2:**
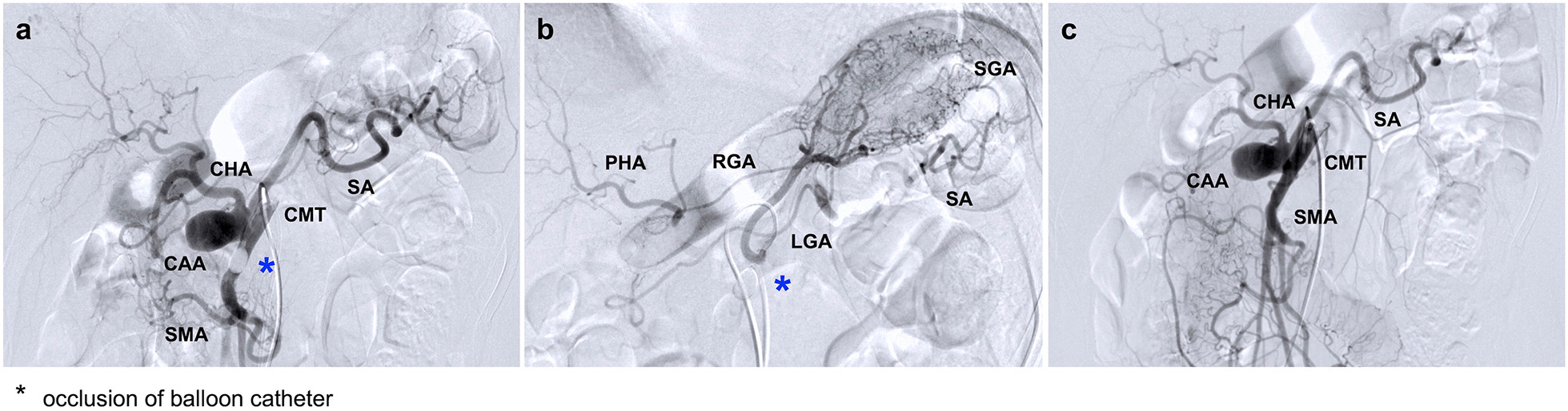
Selective visceral angiography findings.(**a**): Angiography of a celiac artery (CA) with balloon occlusion of SMA. There were no direct branches from the CA to the pancreas. (**b**): Angiography of a left gastric artery (LGA), which originated from the aorta directly, under balloon occlusion of CMT. Collateral circulation from the LGA to splenic artery (SA) via the short gastric artery (SGA) and from the LGA to the proper hepatic artery (PHA) via the right gastric artery (RGA) were observed. (**c**): Angiography findings of the CMT. Under local anesthesia, selective visceral angiography was performed using the bilateral femoral arteries approach. The size of the angiographic catheter was 4 Fr, and a 5 Fr balloon catheter (Selecon MP Catheter -II, Terumo Clinical Supply) was used. The Artis zee (Siemens) angiographic system was used, with ‘Press Duo’ the injection system (Nemoto Kyorindo Co., Ltd, Japan)

Selective visceral angiography was performed to evaluate the visceral circulation of each branch, especially that of the CA and SMA. Angiography revealed that the branches from the CA were only the common hepatic artery (CHA) and splenic artery (SA). We found no discernible microbranches originating directly from the primary CA duct (Fig. 2a). The left gastric artery (LGA) originated from the aorta. Angiography of the LGA with balloon occlusion of the CMT revealed collateral circulation to the proper hepatic artery (PHA) via the right gastric artery (RGA) and to the SA via the short gastric artery (SGA) (Fig. 2b), suggesting that hepatic circulation could be maintained even after occlusion of the CHA.

Based on these anatomical findings, we determined that the safety landing zone for stenting of the CA was not sufficient. In addition, coil embolization of the CAA itself posed a potential risk of coil migration, which could lead to severe complications. Because of the patient's relatively young age, preserving the anatomical antegrade visceral circulation was considered more important than pursuing a less invasive approach through endovascular therapy (EVT). Regarding open surgical procedures, several options are available, such as aneurysmectomy, aneurysmorrhaphy, aortoceliac bypass, aortohepatic bypass, or ligation [4, 5]. Based on CT and angiography findings, simple CAA resection would suffice and be a less invasive surgical procedure for open surgery; therefore, direct CAA resection was performed.

**Fig. 3:**
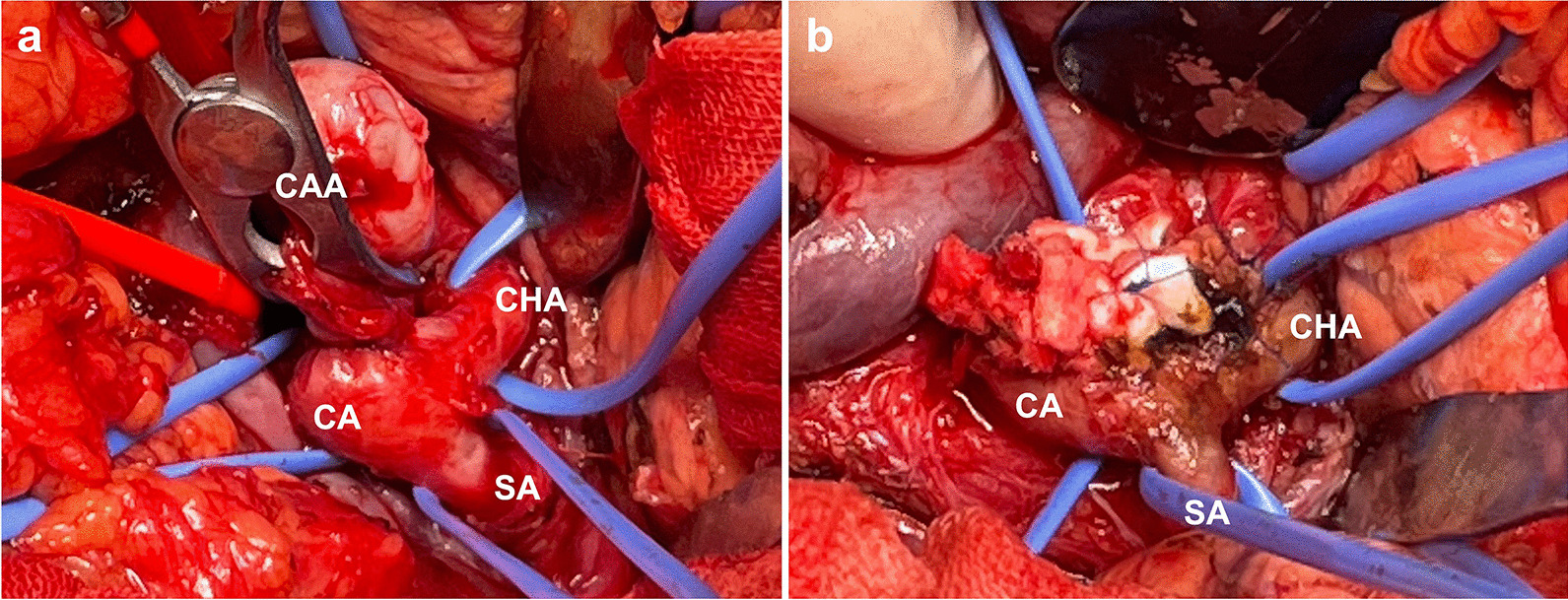
(**a**): The celiac artery aneurysm (CAA) was located near the distal bifurcation of celiac artery (CA). (**b**): Reconstructed CA after the resection of CAA

After the induction of general anesthesia, the abdomen was opened through a midline incision. The pancreas was exposed through an incision in the omentum. Since the CAA was located behind the pancreas on preoperative CT, the CAA was approached with caution to separate it from the pancreas. The portal vein, CMT, CHA, and SA were eventually exposed (Fig. 3a). Following systemic heparinization, direct resection of the CAA and subsequent reconstruction of the CA were performed after clamping the CHA, SA, and proximal CA (Fig. 3b). The total operative time was 249 min, and blood transfusion was not required.

**Fig. 4:**
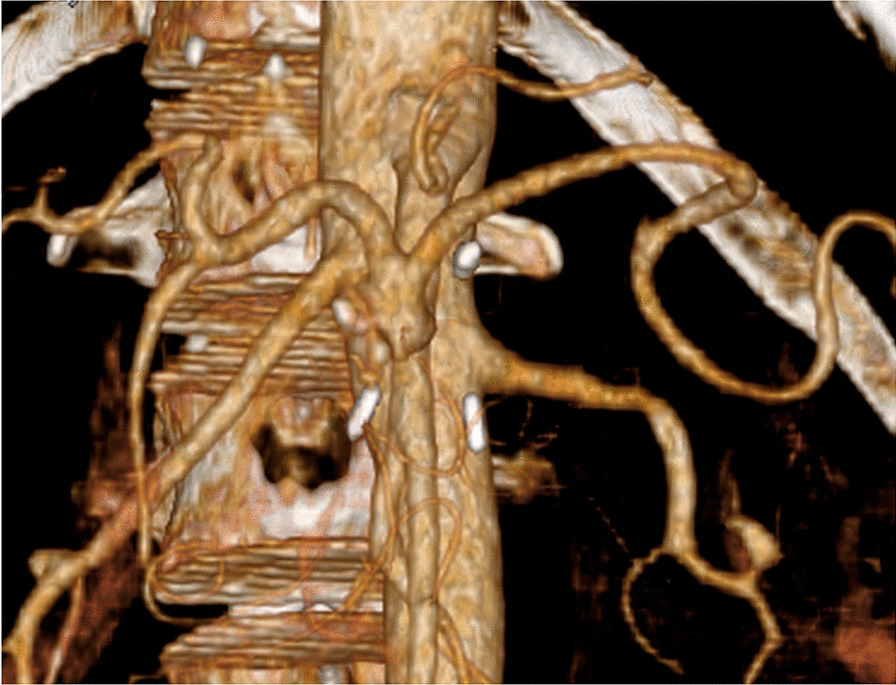
Postoperative enhanced computed tomography findings. The celiac artery is anatomically reconstructed after celiac artery aneurysm resection

**Fig. 5:**
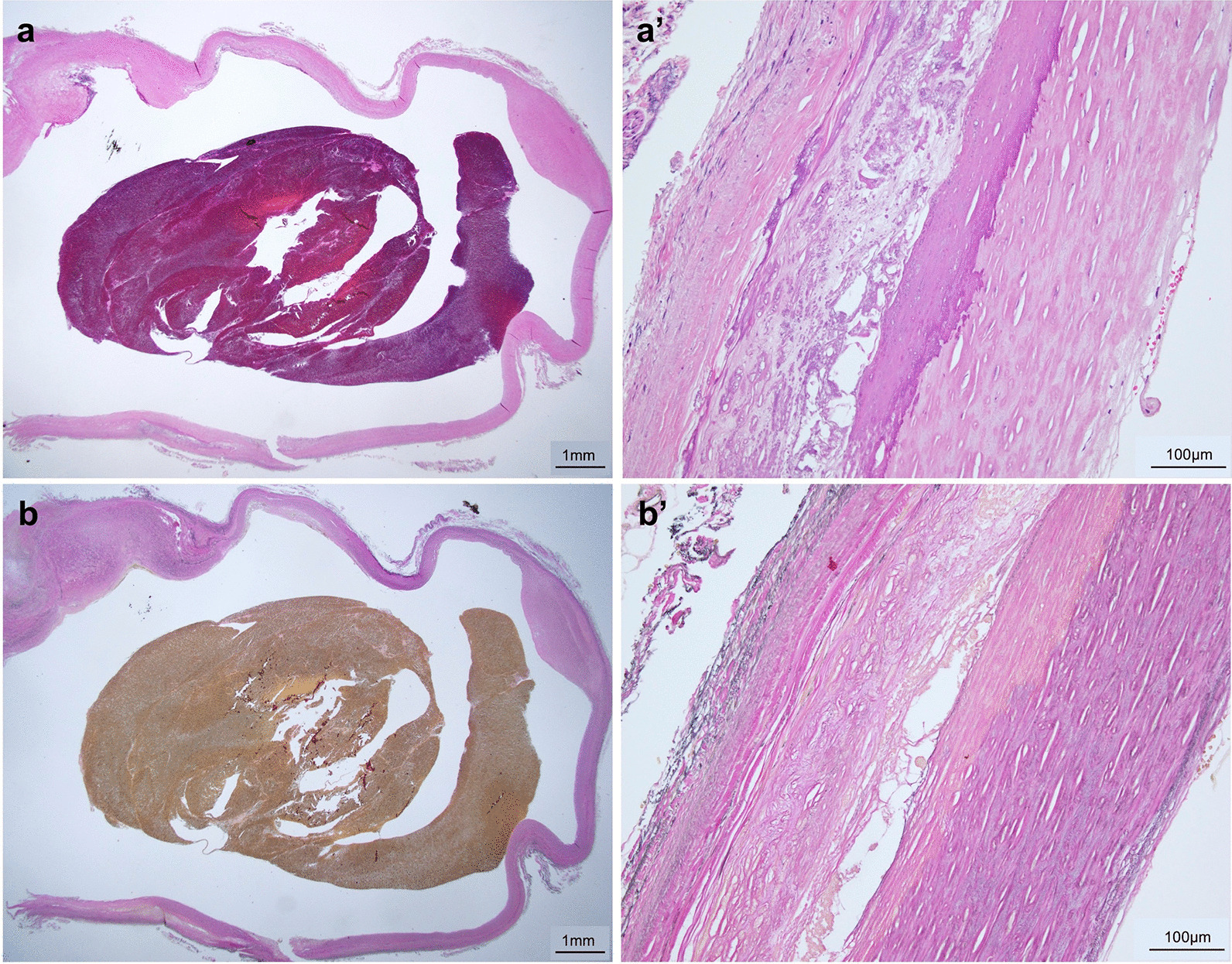
Histological analysis of the aneurysmal wall. (a, a’): hematoxylin-eosin staining (1.25x, 20x, and scale bar = 1 mm, 100 μm, respectively). (b, b’): The existence of an elastic fiber was evaluated by Elastica van Gieson staining (1.25x, 20x, and scale bar = 1- mm, 100 μm, respectively), with no evidence of an absent elastic fiber

On postoperative enhanced CT, the visceral circulation of the CA and SMA was preserved (Fig. 4). Histological analysis of the CAA revealed degenerative true aneurysm, with no evidence of abnormalities, such as lack of elastic fibers or cystic medial necrosis (Fig. 5). The patient was discharged uneventfully on postoperative day 10. A follow-up CT one year later demonstrated no sign of recurrence of the CA or the development of new visceral aneurysms.

## Discussion and conclusions

In patients with CMT, there are various patterns of visceral circulation associated with CA and SMA [1, 2]. In patients with CAA complicated by CMT, precise evaluation of each branch of the visceral circulation is crucial for the planning of the surgical strategy. In the present case, selective visceral angiography demonstrated that there were no direct collaterals from the CA to the pancreas (Fig. 2a), and that there were some collaterals to the CHA and SA via the LGA (Fig. 2b). Given these findings, one potential surgical option could involve EVT such as coil embolization of the CA combined with implantation of a covered stent between the CMT and SMA; this approach aims to maintain distal perfusion of the CA while mitigating the potential risk of coil migration toward the SMA.

The efficacy of EVT in managing visceral artery aneurysms has been reported in various studies [5–8]. Nevertheless, it is essential to acknowledge the significant recanalization rate after successful EVT [5–7], as well as the potential for reperfusion and rupture after successful embolization, indicating that EVT may not be the definitive treatment in all cases. Since the patient was relatively young, a radical open procedure was more desirable than a less invasive EVT procedure. Therefore, open surgery was initially performed in our patient.

The anatomical findings by the visceral angiography also clarified that simple ligation of the CA could be acceptable when accidental bleeding from the CAA cannot be controlled during radical therapy. Conversely, even with advancements in anastomotic aneurysmal procedures, EVT remains a more favorable surgical option due to its minimally invasive nature, simplicity, and reduced risk of accidental bleeding attributed to strong tissue adhesion. Thus, selective visceral angiography can greatly benefit physicians by providing detailed anatomical information on each visceral circulation. Given these advantages, we recommend that selective visceral angiography be performed preoperatively in patients who have CAA with the CMT.

In conclusion, preoperative selective visceral angiography offers valuable insights for planning surgical strategies in patients who have CAA with the CMT by facilitating a more precise analysis of visceral artery circulation.

### Supplementary Information

**Additional file 1**. Supplemental data: the laboratory test.

## Data Availability

All data generated during this study are included in this published article.

## Abbreviations

CMT: Celiacomesenteric trunk
CA: Celiac artery
CAA: Celiac artery aneurysm
SMA: Superior mesenteric artery
CT: Computed tomography
CHA: Common hepatic artery
SA: Splenic artery
LGA: Left gastric artery
RGA: Right gastric artery
SGA: Short gastric artery
EVT: Endovascular therapy
