# Supplementary figures and images for: Use of selective visceral angiography in surgical strategy planning for celiac artery aneurysm in the celiacomesenteric trunk
Source: J Cardiothorac Surg. 2024 Jan 19;19:11. doi: 10.1186/s13019-024-02483-7 (PMC10797990; doi:10.1186/s13019-024-02483-7)

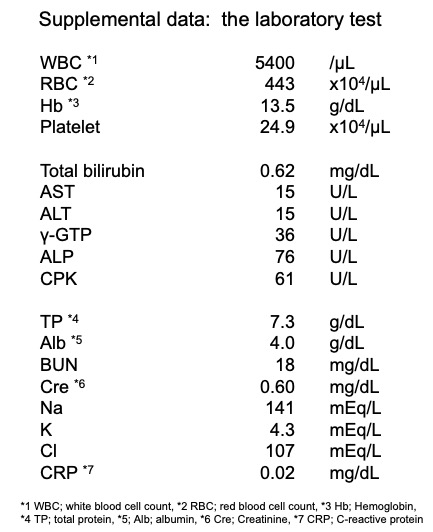

Supplement: Supplementary file 1 — Additional file 1. Supplemental data: the laboratory test. [file 13019_2024_2483_MOESM1_ESM.jpg]
